# Supplementary figures and images for: Inhibition of Asaia in Adult Mosquitoes Causes Male-Specific Mortality and Diverse Transcriptome Changes
Source: Pathogens. 2020 May 15;9(5):380. doi: 10.3390/pathogens9050380 (PMC7281548; doi:10.3390/pathogens9050380)

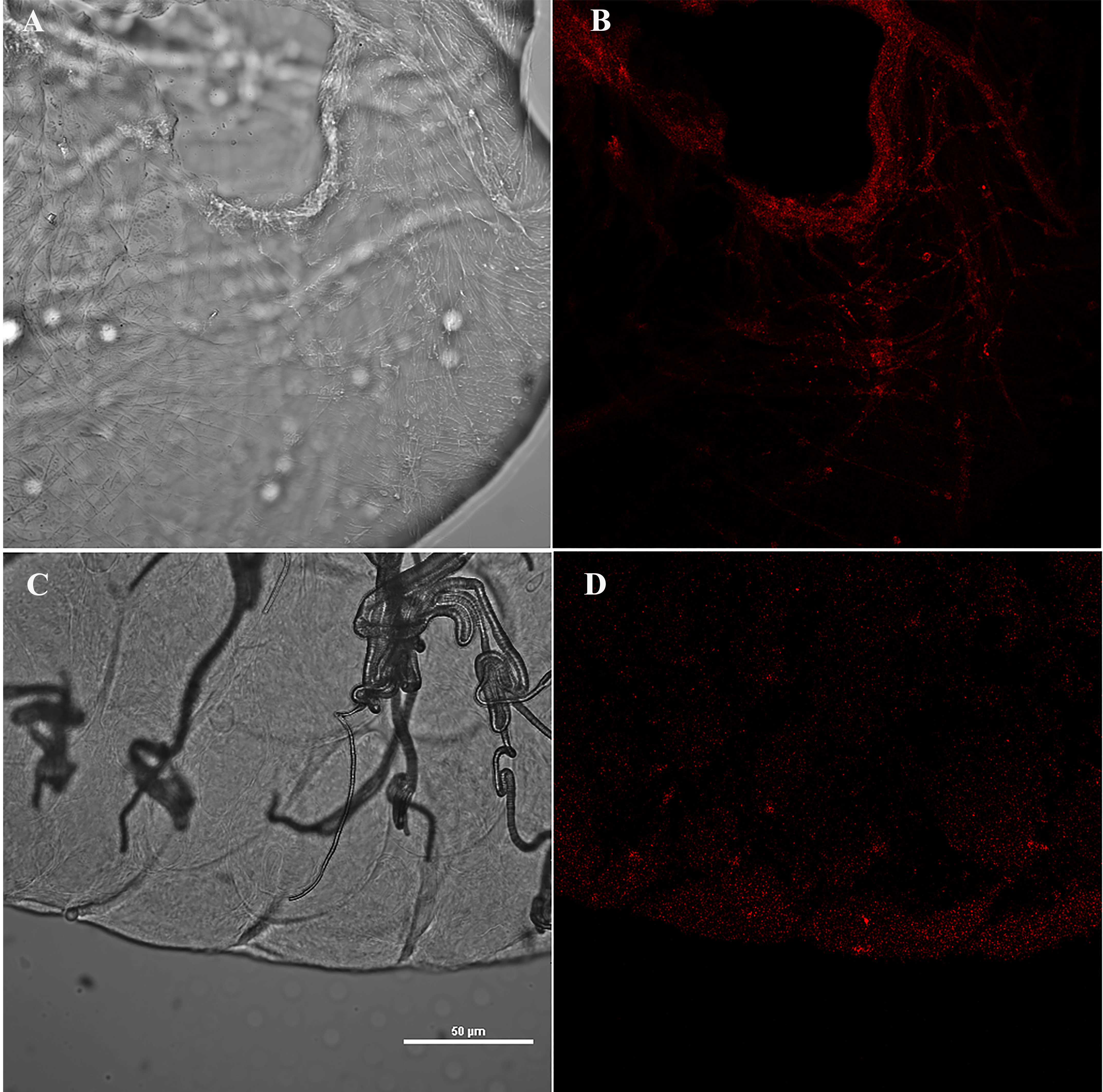

Supplement: Supplementary file 1 [file pathogens-09-00380-s001.zip › SI/Figure S1.tif]

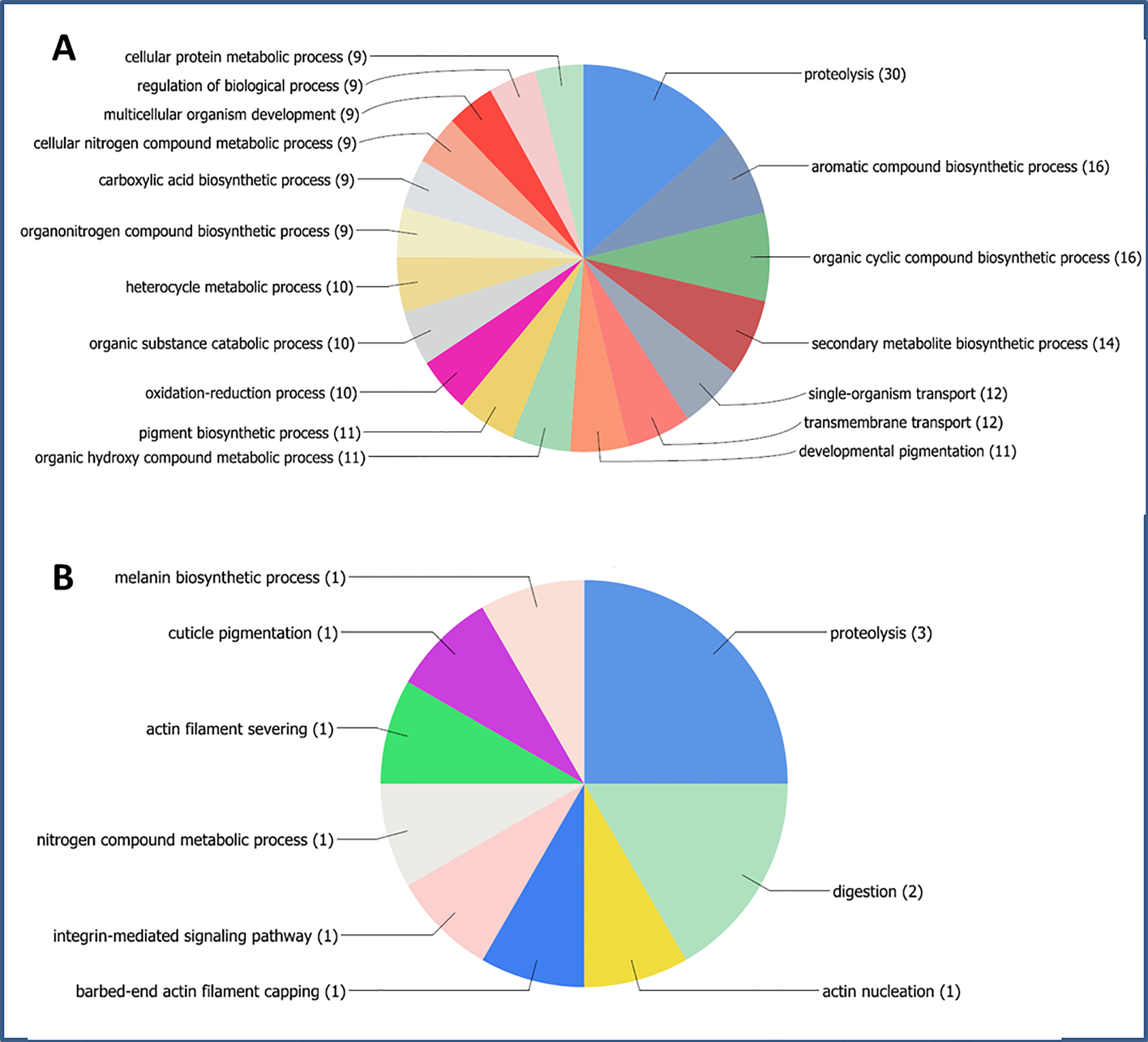

Supplement: Supplementary file 1 [file pathogens-09-00380-s001.zip › SI/Figure S2.tif]
